# The risk of contaminated ultrasound gels in the intensive care unit: lessons from an outbreak of *Burkholderia cenocepacia*

**DOI:** 10.1017/ash.2025.182

**Published:** 2025-07-21

**Authors:** Beevifatimah Ahamed Sha, Paul Anantharajah Tambyah, Shi Poh Li, Sarathamani Rethenam, Lilibeth Silagan Alenton, Ling Chee Poh, Yuan Sun Yong, Peng Sii Yong, Gabriel Yan Zherong, Lasantha Ratnayake, Michelle Ang, Gerald Chua, Surinder Kaur Pada

**Affiliations:** 1 Infection Control Nurse, Ng Teng Fong General Hospital, Singapore, Singapore; 2 Department of Infectious Disease, National University Hospital, Singapore, Singapore; 3 Department of Laboratory Medicine, National University of Hospital, Singapore, Singapore; 4 Department of Laboratory Medicine and Pathology, Woodlands Health, Singapore, Singapore; 5 National Public Health Laboratory, National Centre of Infectious Diseases, Singapore, Singapore; 6 Chairman of the Medical Board, Ng Teng Fong General Hospital, Singapore, Singapore; 7 Department of Infectious Disease, Ng Teng Fong General Hospital, Singapore, Singapore

## Abstract

**Background:**

*Burkholderia cenocepacia* is an environmental Gram-negative bacterium, resistant to many antibiotics and antiseptics, that can survive in aqueous hospital environments. We investigated an outbreak of *B. cenocepacia* in the intensive care unit (ICU) of Ng Teng Fong General Hospital, aiming to identify the source and prevent further transmission.

**Methods:**

The outbreak was detected after two ICU patients developed *B. cenocepacia* bacteremia. Environmental samples, including ultrasound gels, and disinfectants, were collected. Whole genome sequencing (WGS) was used to determine clonality between clinical and environmental isolates. Immediate actions were taken, including a recall of ultrasound gel batches and the use of sterile gel sachets for high-risk procedures.

**Results:**

Ultrasound gels from opened and unopened bottles from multiple hospital areas, including ICU and Radiology, were found to be contaminated with *B. cenocepacia*, with a specific batch (Brand A) linked to the outbreak. WGS analysis confirmed the genetic relatedness of clinical and environmental isolates. A hospital-wide recall of affected gel batches was implemented. Through our regional networks, notification of countries in our immediate region along with alerting our local health authorities for further investigation was also undertaken. Additionally, we continued surveillance of gels and identified further contaminated products.

**Conclusions:**

This outbreak highlights the risks of contaminated medical products, specifically ultrasound gels. Effective environmental sampling, rapid identification, and clear communication with health authorities were key to controlling the outbreak. We have since revised our protocols to mandate the use of sterile gel for invasive procedures and continue monitoring for potential contamination in ultrasound gels.

## Background

*Burkholderia cenocepacia* is an environmental Gram-negative bacteria that is part of the *B.cepacia* complex and resistant to many antiseptics and antibiotics.^1,2^ It can survive in aqueous environments, including antiseptic solutions in the healthcare environment.^3,4^ Previous reports have demonstrated transmission from contaminated liquids or moist environmental surfaces in hospital settings in association with contaminated mouthwash, intravenous saline, liquid docusate, anesthetic gel, and ultrasound gel.^1,3,5–8^

Ng Teng Fong General Hospital is an integrated facility with an acute care hospital (700 beds, up to 75 ICU beds) and a community hospital (400 beds). We recently investigated an outbreak of *B. cenocepacia* complex infections that occurred in our ICU.

## Description of outbreak

Our microbiology laboratory informed the Infection Prevention Team on 18^th^ August 2021 of two patients from the intensive care unit who had blood cultures positive for *Burkholderia cenocepacia*. The first blood culture was identified on 26^th^ July 2021 and the second on the 16^th^ August 2021 from patients nursed in different rooms and parts of the ICU as shown in (supplementary material in Figure S1), a schematic map visualization of the layout of the ICU divided into “Pods” in operation during this outbreak. An outbreak investigation was started by the Infection Prevention team following the laboratory alert.

Cases were defined as any patient currently or recently hospitalized in the ICU with a blood culture positive for *B. cenocepacia* complex between 26th February 2021 to 31^st^ August 2021. No additional cases were found. There had never been a *B. cenocepacia* bacteremia reported from this facility. Additionally, there were no changes in microbiological testing practices or reporting protocols.

Case 1 was a 74-year-old male who was admitted on 16^th^ July 2021 to ICU for bilateral empyema with mediastinitis secondary to an esophageal perforation. A central venous catheter (Right Femoral) was inserted on the 17^th^ July 2021. The patient had blood drawn for blood culture which was positive on 26^th^ July 2021, 11 days post admission. The patient was eventually discharged well on 31^st^ December 2021.

Case 2 was a 63-year-old female admitted on 6^th^ August 2021 to ICU for severe gram negative sepsis leading to purpura fulminans. A central venous catheter (Right Internal Jugular) was inserted on 6^th^ August 2021. The patient had a blood culture drawn which was positive on 16^th^ August 2021, 11 days post admission. The patient succumbed to her underlying illness and passed away on 23/8/2021.

## Laboratory methods

All samples were sent to our hospital microbiology laboratory. All positive samples were then subsequently sent on to the National Public Health Laboratory which is the national reference center for whole genome sequencing (WGS). Environmental samples were gel – plated on sheep blood agar and incubated in ambient air at 35^0^ C for 48 hours. Patient samples were plated on Sheep Blood, Chocolate and MacConkey agar and incubated in 5% CO_2_ at 35^0^ C for 48 hours. Whole genome sequencing was susequently performed. WGS libraries were generated using Nextera XT DNA Library Preparation Kit and sequenced using Illumina MiSeq System as described in reference 9.

## Investigation

As *B. cenocepacia* is a known environmental organism, the investigation focused on potential environmental sources in the ICU in addition to routine outbreak investigations. We collected environmental culture samples from opened mouthwash bottles, opened disinfectant (hexanol®) solution, tap water and surface swabs from the sinks, opened packets of antiseptic body wipes, ultrasound probes, opened ultrasound gel bottles, and bed rails.

We performed a line listing (found in supplementary material Figure S2), to determine if we could identify any significant common risk factors between the infected patients.

As endoscopy and the operating theater (OT) appeared to be areas where infection might have occurred, we also collected samples from there with a focus on aqueous sources and included Brand A bottle (new), Storz ® Endoscope, Storz ® Endoscope Probe, OT sedation port, unopened sterile gel (Brand B), Heat and Mechanical Exchange filters in the operating room, unopened chlorhexidine bottles, unopened Cetrimide® disinfectant solutions, and unopened Sterile water containers. While the same operating theater was used for both patients, they underwent different procedures, were attended by different anesthetists and surgeons, and due to our strict policy of ‘single use, single patient’ for all IV medications, had not shared any fluids or drugs. Additionally, during our visit to the operating room, we did not find any obvious environmental issues such as condensation or leaks.

We then audited infection control practices related to reprocessing of scopes and investigated any potential lapses that could have contributed to the outbreak.

## Results

We discovered that earlier in the year, two endoscopes (one Colonoscope and one Gastroscope) had tested positive for *B. cenocepacia* during routine endoscopy surveillance. These scopes were sent back to the vendor for repair, re-cultured, and found to be negative before being used for other patients. They continued to test negative during routine surveillance cultures after returning from the vendor.

We found positive growth of *B. cenocepacia* from ultrasound gels we had sampled from the ICU. We then expanded ultrasound gel sampling to include all areas within the hospital where this gel was used, including the Materials Management Department (MMD) storage area, Emergency Department, radiology suite, urology, cardiology clinics, and neurodiagnostic clinics. We sent both opened and unopened bottles of gel for microbiological sampling.

All positive cultures were sent to the National Public Health Laboratory for WGS to confirm species identity and support outbreak investigation findings via molecular typing. Briefly, isolates were sequenced as described previously and WGS analysis ran using the Nullarbor bioinformatics pipeline.^9,10^

The environmental culture samples collected on 19 August 2021 from ICU POD 1 and 2 were all negative for *B. cenocepacia* except for the three used “Brand A” bottles from ICU. A total of 12 samples were positive on culture from opened and unopened ultrasound gels in the MMD, Emergency Department, radiology suit, cardiology lab and urology clinic. The details are in Table 1.

**Table 1.** Clinical and environmental isolates

S/N	NHPL no	Name	Collection date	Submitting Lab	Sample Source	Lot Number	Species	*in silico* MLST
1	OTH-21-0020	ZBB	16/8/2021	NTF	Blood	–	*B. cenocepacia*	ST241
2	OTH-21-0021	RBJ	8/3/2021	NTF	Blood	–	*B. cenocepacia*	New ST
3	OTH-21-0022	TPP@TCP	26/7/2021	NTF	Blood	–	*B. cenocepacia*	ST241
4	OTH-21-0023	–	19/8/2021	NTF	Brand A	Lot 1	*B. cenocepacia*	ST241
5	OTH-21-0024	–	19/8/2021	NTF	Brand A	Lot 1	*B. cenocepacia*	ST241
6	OTH-21-0025	–	20/8/2021	NTF	Brand A	Lot 1	*B. cenocepacia*	ST241
7	OTH-21-0026	–	23/8/2021	NTF	Brand A	Lot 1	*B. cenocepacia*	ST241
8	OTH-21-0027	–	23/8/2021	NTF	Brand A	Lot 1	*B. cenocepacia*	ST241
9	OTH-21-0028	–	23/8/2021	NTF	Brand A	Lot 1	*B. cenocepacia*	ST241
10	OTH-21-0029	–	23/8/2021	NTF	Brand A	Lot 1	*B. cenocepacia*	ST241
11	OTH-21-0030	–	23/8/2021	NTF	Brand A	Lot 1	*B. cenocepacia*	ST241
12	OTH-21-0031	–	19/8/2021	NTF	Brand A	Lot 2	*B. cenocepacia*	ST241

Brand A: Isolates from sampled ultrasound gels.

On the afternoon of 20 August 2021, the laboratory informed us that two cultures of the ultrasound gel “Brand A” used in the ICU had been preliminarily positive for *B. cenocepacia*. To exclude the possibility of a batch contamination of ultrasound gel, we subsequently sent unopened bottles of “Brand A” from the ICU and OT for culture. At that point, we also sent samples of other brands of ultrasound gels used in our hospital for culture.

**Figure 1.**
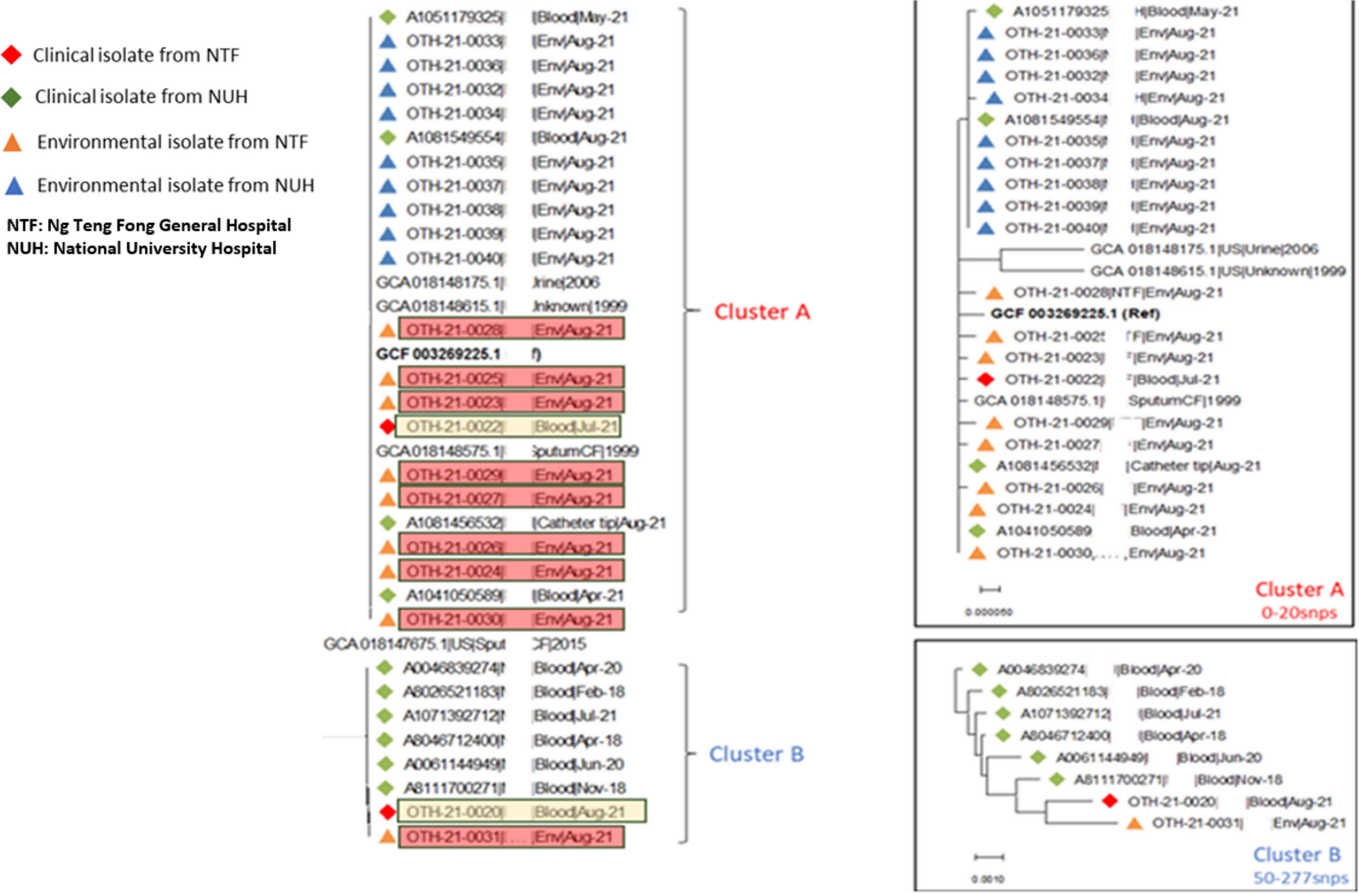
Maximum likelihood phylogenetic analysis of 30 ST241 *B. cenocepacia* isolates.

Whole genome sequencing showed that all clinical and environmental isolates were genetically related (<5 snips), except for one clinical and one environmental isolate (Refer to Figure 1). We promptly alerted other hospitals in Singapore who may have been using the same brand of gel. Subsequently, another hospital within our cluster using the same brand of gel reported positive samples. The phylogenetic tree in Figure 1 details the results of these samples from both hospitals implicated in this outbreak from patients as well as from the gels (labeled as environmental isolates).

## Immediate mitigation actions taken

Upon receiving the first preliminary result indicating contaminated opened bottles of ultrasound gel, an immediate stop order was issued to the ICU for the use of “Brand A.”

We informed our MMD, responsible for procuring and storing hospital supplies, of the ultrasound “Brand A” contamination and instructed them to source for alternatives. Soon after, the lab confirmed one further positive *B. cenocepacia* sample from an ICU ultrasound gel bottle.

Whilst investigations were ongoing, we directed high-risk areas (OT, ICU, intervention radiology, and Kidney Unit) to use single sachet sterile gel for all ultrasound procedures. We also emphasized the importance of hand hygiene, environmental cleaning, and equipment disinfection.

Other areas, including clinics (cardiology, urology, and neurodiagnostic laboratory) and general wards, were provided with an alternative gel brand available on site (Brand B). To determine if there was a problem unique to “Brand A,” 10 unopened bottles with different lot numbers were sent from various locations including the Emergency Department, Interventional Radiology Suite, Community Hospital, and Specialist Outpatient Clinics. Five of the ten unopened bottles from the same batch were positive for *B. cenocepacia*. As this appeared to be a batch contamination of one lot of ultrasound gel (Brand A), we issued a hospital-wide recall for all “Brand A” bottles, replacing them with the alternative brands that were in stock within the hospital.

Additionally, the Head of our Infection Prevention Team alerted the heads of other public hospitals in Singapore through email. Only one other hospital found “Brand A” contamination at their site as described above. Through regional contacts, we also informed neighboring countries, who did not report any contaminated gels to us.

In tandem, we alerted the Health Science Authority (HSA) and the Ministry of Health (MOH) as this might have had far-reaching consequences. As part of the measures taken, HSA and MOH issued an advisory via email to all hospitals in Singapore on the 30 August 2021 with an attached letter from the manufacturer to cease use of all batches and lots of the gel, advising all hospitals to stop using affected products and ensure removal from all clinical areas.^11^ They recommended a high index of suspicion for *B. cenocepacia* complex group infections in patients who had had ultrasound-guided procedures using these gels. They also recommended that hospital managers remind all hospital staff in ICU, Radiology, and Catheterization Laboratories to use only sterile gel for all invasive procedures. They were also asked to consider sending any unopened ultrasound gel bottles from manufacturers not listed in their circular for culture to eliminate contamination risks. In case of new findings, the hospitals were instructed to notify MOH and HSA who would pre-emptively institute a nationwide recall while investigations were underway if necessary.

During our own audits of practices in the ICU, we discovered that the ICU had used bottled non-sterile ultrasound gel to identify the most appropriate insertion site for Central venous catheter placement prior to insertion. Whilst aseptic technique was then used for insertion, high bioburden and inadequate cleaning of the skin may have contributed to the lines becoming infected and causing the *B. cenocepacia* bacteremia in our two ICU patients. A policy review on such procedures was undertaken involving all the stakeholders which was refined to clarify that for intravascular access procedures and procedures that may involve mucous membrane contact, sterile gel should always be used.

## Continuous surveillance testing

**Figure 2.**
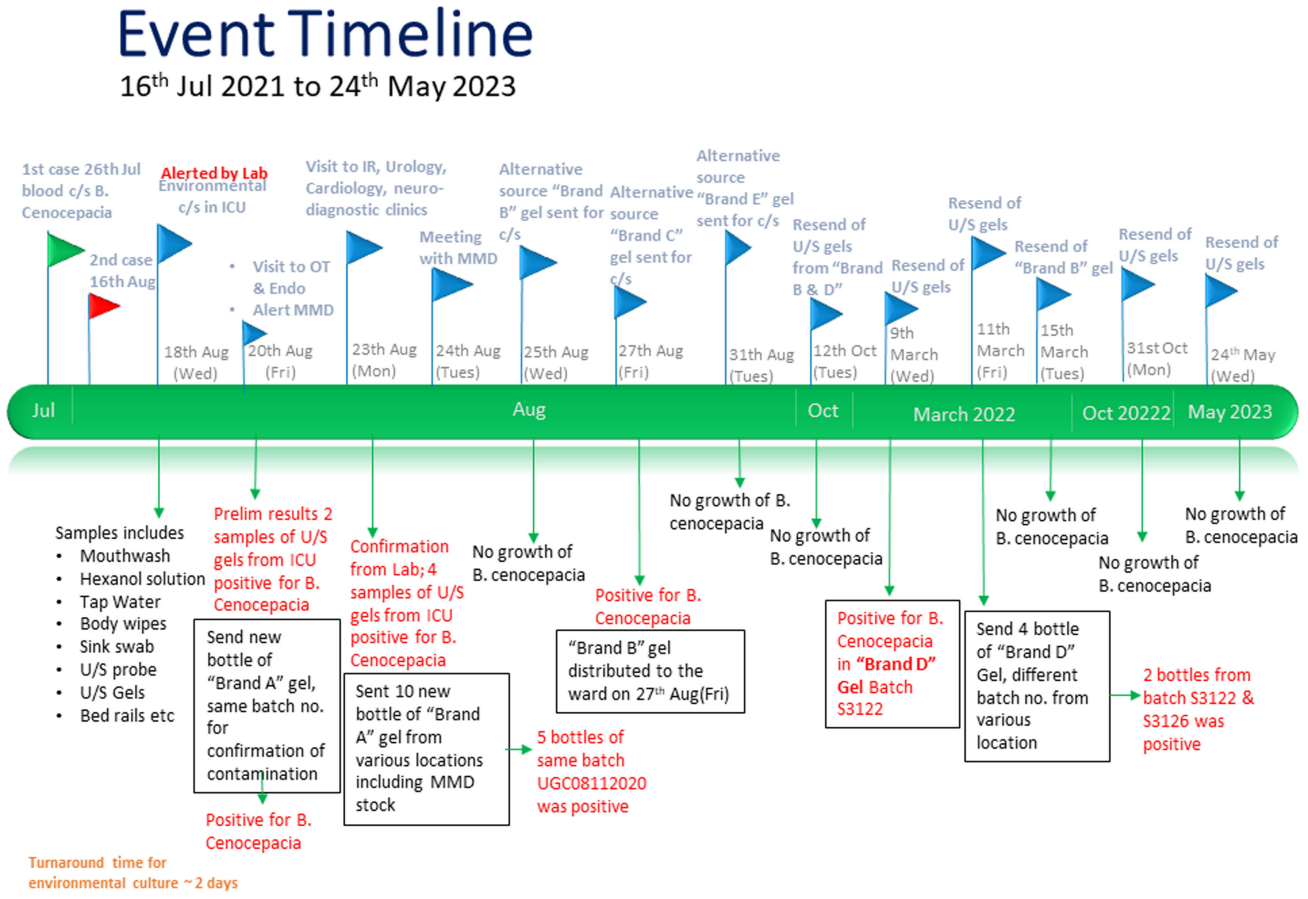
Event timeline.

Between 25th August 2021 and 31st August 2021, MMD sourced alternative ultrasound gel providers. Samples of unopened bottles of four alternative brands were sent for culture, and out of the four sample bottles, “Brand C” gel also tested positive for *B. cenocepacia*. Given the propensity of these gels to contamination, we decided, to conduct surveillance sampling on a quarterly basis. Figure 2 summarizes the event timeline as described in other sections above.

After one round of negative cultures from unopened ultrasound gel bottles, our surveillance testing on 9th Feb 2022 identified that “Brand D,” lot S3122, tested positive for *B. cenoepacia*. Additional testing of five unopened bottles of the same brand from various lot numbers revealed that three from batch S3122 and batch S3126 grew *B. cenocepacia*. We immediately communicated these results to MMD, recalled the affected batches from the wards, and sent emails to various affected departments.

## Discussion

This outbreak highlighted the importance of environmental and product sampling in investigating healthcare-associated outbreaks. Our surveillance and molecular epidemiological investigation suggested a batch contamination. There have been many studies reporting intrinsic batch contamination and B cepacia outbreaks. The details of some of these can be found in Table 2.

**Table 2.** Characteristics of B Cepacia outbreaks and infections in healthcare settings

First author/ year	Transmission source	Patient population	Type of infection	Study type
Shaban RZ 2017^1^	contaminated gel packaged in sachets for use within the sterile ultrasound probe cover.	ICU	9 blood culture, 1 abscess, 1 ascitic and 1 urine	Outbreak: strong causation 11 patients across 4 hospitals in Australia all same multilocus sequence type
Angrup A 2022^2^	Contaminated Ultrasound gel: 8 intrinsic contamination from manufacturing site 4 extrinsic contamination from environmental source	8 ICU, 4 wards, 3 unknown (some outbreaks involved both wards and ICU)	10 studies; bacteremia most common 4 studies; Urinay tract infection	Systematic review was conducted to analyze Bcc outbreaks due to ultrasound (US) gel 14 studies included Strong causation by molecular typing undertaken
Bilgin H 2021^3^	Intrinsic contamination of 2% chlorhexidine (CHG) mouthwash solution	ICU	Respiratory cultures in ventilated patients	Outbreak Strong causation 6 patients
Marquez L 2017^5^	Intrinsic contaminated liquid docusate	Pediatric ICU	18 respiratory tract cultures, 5 blood cultures, 4 urine cultures, and 3 stool cultures (some patients had positive cultures from multiple sites)	Outbreak Strong causation 24 patients
Solaimalai D 2019^6^	Extrinsic contamination of Ultrasound Gel	Pediatric ICU	Bacteremia	Outbreak: 7 cases strong causation
Häfliger E 2020^7^	Medical device 19 (17.1%) Medical preparation (including solutions, drugs, disinfectants) 66 (53.2%) Intrinsically contaminated products 35 (28.2%) Extrinsically contaminated products 55 (44.4%) Unknown if intrinsically or extrinsically contaminated product 34 (27.4%)	Number of outbreaks with ICU involvement 65 (58.6%) Number of outbreaks occurring exclusively in ICUs 39 (35.1%)	Not available	Systematic review 111 studies identified. Clonal relationship of strains was proven positive in 52.2%, negative in 14.4% and remained undetermined in 32.4%.

Study type: each article’s definition of “case series (multiple)” or “outbreak” or “systematic review” was used.

“Strong causation” was added when at least 1 molecular typing method indicated relatedness to a contaminated product.

Upon detailed interviews with the ICU staff looking after both these patients and review of the practices in ICU, we hypothesized that the transmission occurred during central line insertion. As a preliminary step in mapping a suitable site for central venous catheter insertion, brand bottle A non-sterile gel was used. Although this was wiped off, the disinfection procedure and line insertion that followed did not allow for sufficient contact time such that there was still a significant bioburden of *B cenocepacia* at the insertion site at the time of central line insertion. Different bottles of Brand A were used for both patients, however, they were of the same batch number which is most likely due to a product contamination of the batch. Through interaction with the local regulatory agencies and with the other hospitals in the country, we were able to alert other clinicians and potentially prevent other infections through the recall of the product. Additionally, the HSA notified the vendor (Pan-Malayan Medical Hypermart) that all batches and lots of the products, as well as the ultrasound gel and ECG gel (Brand A), should be ceased from use immediately and tested for *B. cenocepacia*. They also assisted in alerting users in the country to discontinue using Brand A. We also identified other contaminated ultrasound gel products which highlighted the propensity of such contamination in these products. The early detection from surveillance allowed us to prevent these products being used on patients.

We also immediately implemented the use of single-use sterile gel sachets for ultrasound-guided percutaneous procedures as has been recommended by others.^12^ Even though policies including ours recommend the use of sterile gel in invasive procedures, this is not always adhered to. Clinicians doing preparatory ultrasound scanning of the area before insertion of CVCs often use non-sterile gel believing that subsequent disinfection of the skin should be able to deal with any contamination that may occur from non-sterile gel. Unfortunately, biofilm formation by *B. cepacia* complex organisms is well known, and when the bioburden is high, strict aseptic preparation methods may not suffice especially because these organisms are often resistant to antibiotics and antiseptics. This issue is compounded by the fact that many procedurists rarely wait for the contact times required for disinfectants to act optimally and often do not wipe off all the non-sterile ultrasound gel as recommended.^13^ These strategies of surveillance testing of gels as well as using sterile gel for high-risk procedures have helped prevent further infections; we have had no further cases to our knowledge.

We also wish to highlight the importance of outbreak response activities, rapid notification systems, and information dissemination both with national regulators and also regionally as these products have widespread distribution. This strategy has been deployed elsewhere and was instrumental in bringing outbreaks of *B. cepacia* group under control.^2^

A key concern is how to make these gels safe given that it may not be possible to completely eliminate the use of non-sterile gels in ultrasound procedures. In an exploratory study in Malawi, chlorhexidine and alcohol were added to disinfect the gel prior to ultrasound for 20 patients. Although there was some modest success, this intervention was not found to be practical on a large scale. In addition, as these products were being compounded locally, the authors were fearful that such manipulation of the gel may lead to other issues downstream.^14^ In another in vitro study that looked at cleaning the tip of gel bottles after use with a disinfectant cloth, there was a reduction but not elimination of bacterial contamination of either the bottle or the ultrasound probes.^12^

Several ultrasound gel brands and batches were tested during our investigation of this outbreak, and we have continued to carry out surveillance testing and audits on these gels. As we have continued to find contaminated unopened bottles of gel even after the outbreak, we have now shifted our policy to mandate that all ICU procedures requiring ultrasound should use sterile gel. However, these come in small volume sachets which may not be adequate for many ICU procedures. This is clearly something manufacturers should address. A risk stratification approach based on the intactness of the skin during and immediately after the ultrasound procedure could be considered in deciding when the use of single sterile ultrasound gel sachets needs to be implemented strictly to prevent the spread of infection. To complicate matters further, in addition to ultrasound gels, other aqueous solutions and gels have also been reported as sources of *B. cepacia* group outbreaks.^3,5,7^ Education and reminders to staff to encourage compliance to policy is probably the only way to ensure safe use of these gels.

## Conclusion

In summary, we were fortunate that our microbiology laboratory alerted us when two blood cultures flagged positive for an unusual organism known to be associated with outbreaks in health care settings. The main take-home messages from our experience are that contaminated medical products can be a source of outbreaks, emphasizing the need for rigorous testing and surveillance as well as ensuring appropriate use of non-sterile products in clinical settings. Rapid response and coordination are crucial in containing outbreaks and preventing further infections on a wider scale and that prevention strategies are key to patient safety, highlighting the importance of implementing policies and education to minimize the risk of outbreaks.

## Supporting information

10.1017/ash.2025.182.sm001 Ahamed Sha et al. supplementary material 1 Ahamed Sha et al. supplementary material

10.1017/ash.2025.182.sm002 Ahamed Sha et al. supplementary material 2 Ahamed Sha et al. supplementary material

## Supplementary material

The supplementary material for this article can be found at https://doi.org/10.1017/ash.2025.182

## References

[1] Shaban RZ , Maloney S , Gerrard J , et al. Outbreak of health care-associated *Burkholderia cenocepacia* bacteremia and infection attributed to contaminated sterile gel used for central line insertion under ultrasound guidance and other procedures. Am J Infect Control 2017;45:954–958. doi: 10.1016/j.ajic.2017.06.025 28757084

[2] Angrup A , Kanaujia R , Biswal M , Ray P . Systematic review of ultrasound gel associated Burkholderia cepacia complex outbreaks: clinical presentation, sources and control of outbreak. Am J Infect Control 2022;50:1253–1257. doi: 10.1016/j.ajic.2022.02.005 35158013

[3] Bilgin H , Gelmez GA , Bayrakdar F , et al. An outbreak investigation of *Burkholderia cepacia* infections related with contaminated chlorhexidine mouthwash solution in a tertiary care center in Turkey. Antimicrob Resist Infect Control 2021;10:143. doi: 10.1186/s13756-021-01004-8 34629114 PMC8502507

[4] Tavares M , Kozak M , Balola A , Sá-Correia I . *Burkholderia cepacia* complex bacteria: a feared contamination risk in water-based pharmaceutical products. Clin Microbiol Rev 2020;33. doi: 10.1128/cmr.00139-19 PMC7194853 32295766

[5] Marquez L , Jones KN , Whaley EM , et al. An outbreak of *Burkholderia cepacia* complex infections associated with contaminated liquid docusate. Infect Control Hosp Epidemiol 2017;38:567–573. doi: 10.1017/ice.2017.11 28166854

[6] Solaimalai D , Ragupathi NKD , Ranjini K , et al. Ultrasound gel as a source of hospital outbreaks: Indian experience and literature review. Indian J Med Microbiol 2019;37:263–267. doi: 10.4103/ijmm.ijmm_19_249 31745029

[7] Häfliger E , Atkinson A , Marschall J . Systematic review of healthcare-associated Burkholderia cepacia complex outbreaks: presentation, causes and outbreak control. Infect Prev Pract 2020;2:100082. doi: 10.1016/j.infpip.2020.100082 34368718 PMC8335909

[8] Centres for Disease Control and Prevention. Burkholderia Cepacia in Healthcare Settings. https://www.cdc.gov/hai/organisms/bcepacia.html.

[9] Lim AYH , Ang MLT , Cho SSL , Ng DHL , Cutter J , Lin RTP . Implementation of national whole-genome sequencing of *Mycobacterium tuberculosis*, National Public Health Laboratory, Singapore, 2019—2022. Microb Genom 2023;9:001139. doi: 10.1099/mgen.0.001139 38010371 PMC10711301

[10] Tseemann. GitHub - tseemann/nullarbor: :floppy_disk: “Reads to report” for public health and clinical microbiology. GitHub. https://github.com/tseemann/nullarbor

[11] Mullins K , Burnham K , Henricson EK , Cohen S , Fair J , Ray JW . Identification and analysis of bacterial contamination of ultrasound transducers and multiuse ultrasound transmission gel bottle tips before and after the aseptic cleansing technique. J Ultrasound Med 2020;39:1957–1963. doi: 10.1002/jum.15300 32339352

[12] Hudson MJ , Park SC , Mathers A , et al. Outbreak of *Burkholderia stabilis* infections associated with contaminated nonsterile, multiuse ultrasound gel — 10 states, may–september 2021. MMWR Morb Mortal Wkly Rep 2022;71:1517–1521. doi: 10.15585/mmwr.mm7148a3 36454695 PMC9721143

[13] Hudson MJ , Park SC , Mathers A , et al. Outbreak of *Burkholderia stabilis* infections associated with contaminated nonsterile, multiuse ultrasound gel — 10 states, may–september 2021. MMWR Morb Mortal Wkly Rep 2022;71:1517–1521. doi: 10.15585/mmwr.mm7148a3 36454695 PMC9721143

[14] Okere P . Low-cost antimicrobial fortification of ultrasound coupling gel: an ergonomic innovation to combat sonology-acquired nosocomial infections. Malawi Med J 2019;31:45. doi: 10.4314/mmj.v31i1.8 31143396 PMC6526342

