# Supplementary figures and images for: The risk of contaminated ultrasound gels in the intensive care unit: lessons from an outbreak of Burkholderia cenocepacia
Source: Antimicrob Steward Healthc Epidemiol. 2025 Jul 21;5(1):e161. doi: 10.1017/ash.2025.182 (PMC12281230; doi:10.1017/ash.2025.182)

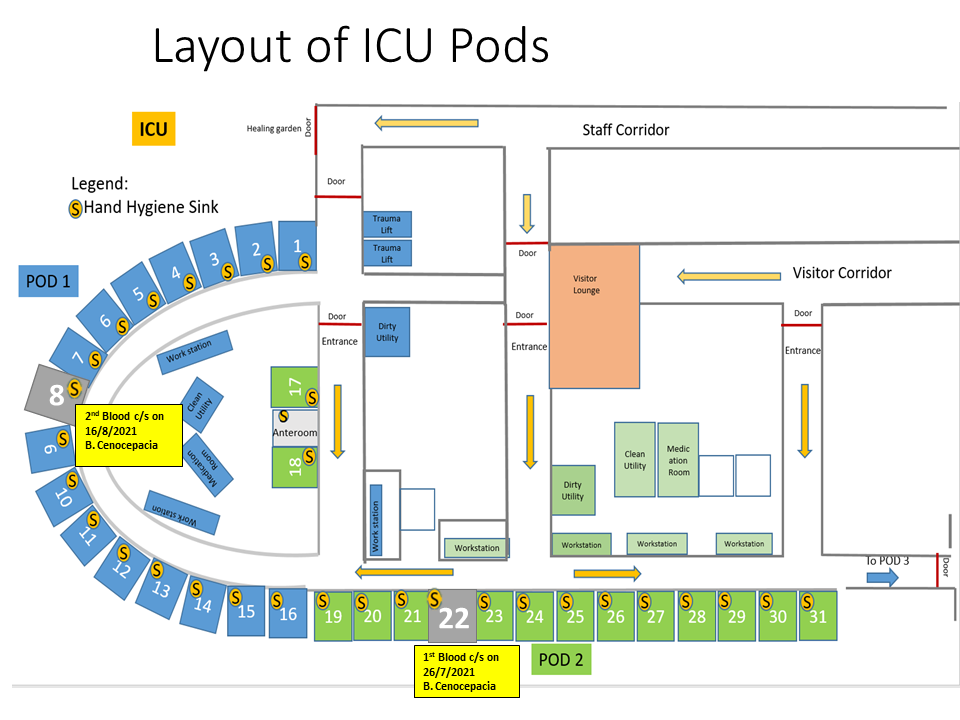

Supplement: Ahamed Sha et al. supplementary material 1 — Ahamed Sha et al. supplementary material [file S2732494X25001822sup001.tif]

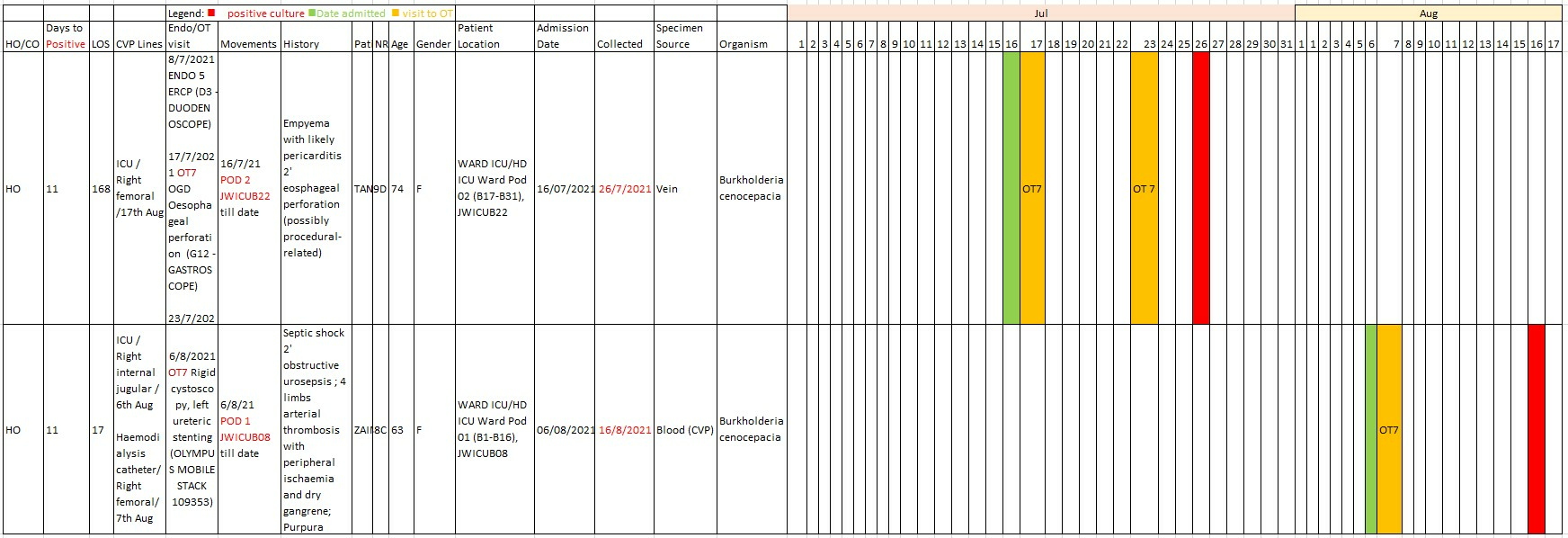

Supplement: Ahamed Sha et al. supplementary material 2 — Ahamed Sha et al. supplementary material [file S2732494X25001822sup002.tif]
